# Perceptions of appropriate treatment among the informal allopathic providers: insights from a qualitative study in two peri-urban areas in Bangladesh

**DOI:** 10.1186/s12913-019-4254-3

**Published:** 2019-06-26

**Authors:** M. Monaemul Islam Sizear, Herfina Y. Nababan, Md. Kaoser Bin Siddique, Shariful Islam, Sukanta Paul, Anup Kumar Paul, Syed Masud Ahmed

**Affiliations:** 1 WaterAid, Dhaka, Bangladesh; 2 Nossal Institute for Global Health, Melbourne School of Population and Global Health, The University of Melbourne, Melbourne, Australia; 3 International Centre for Diarrhoeal Disease Research, Bangladesh (icddr,b), Dhaka, Bangladesh; 4 Institute for Physical Activity and Nutrition (IPAN), School of Exercise and Nutrition Sciences, Deakin University, Geelong, Australia; 5 Terre des hommes Foundation, Dhaka, Bangladesh; 6 mPower Social Enterprises Ltd., Dhaka, Bangladesh; 7 James P. Grant School of Public Health, BRAC University, Dhaka, Bangladesh

**Keywords:** Informal providers, Private sector for health, Health service delivery, Appropriate treatment, Patient safety

## Abstract

**Background:**

How the informal providers deliver health services are not well understood in Bangladesh. However, their practices are often considered inappropriate and unsafe. This study attempted to fill-in this knowledge gap by exploring their perceptions about diagnosis and appropriate treatment, as well as identifying existing barriers to provide appropriate treatment.

**Methods:**

This exploratory study was conducted in two peri-urban areas of metropolitan Dhaka. Study participants were selected purposively, and an interview guideline was used to collect in-depth data from thirteen providers. Content analysis was applied through data immersion and themes identification, including coding and sub-coding, as well as data display matrix creation to draw conclusion.

**Results:**

The providers relied mainly on the history and presenting symptoms for diagnosis. Information and guidelines provided by the pharmaceutical representatives were important aids in their diagnosis and treatment decision making. Lack of training, diagnostic tools and medicine, along with consumer demands for certain medicine i.e. antibiotics, were cited as barriers to deliver appropriate care. Effective and supportive supervision, training, patient education, and availability of diagnostics and guidelines in Bangla were considered necessary in overcoming these barriers.

**Conclusion:**

Informal providers lack the knowledge and skills for delivering appropriate treatment and care. As they provide health services for substantial proportion of the population, it’s crucial that policy makers become cognizant of the fact and take measures to remedy them. This is even more urgent if government’s goal to reach universal health coverage by 2030 is to be achieved.

**Electronic supplementary material:**

The online version of this article (10.1186/s12913-019-4254-3) contains supplementary material, which is available to authorized users.

## Background

Informal health care providers (informal providers) are the providers who engage in health activities for which they do not possess official qualification or permit [1]. Their informality can be identified by their training, business model, registration and regulation as well as professional affiliations [1]. In Bangladesh, the term refers to those who are not registered with any government regulatory body, therefore operate beyond government’s oversight [2]. They comprise of a range of providers including community health workers (CHWs), informal (unqualified) allopathic providers (e.g. village doctors and drugstore sales people/ drug vendors), traditional healers, non-secular faith healers, traditional birth attendants and homeopaths [2]. Generally, they do not receive any training from any recognized medical training institution; however, workshop, seminar, and apprenticeships are their typical learning platforms [2]. CHWs for example, either the ones who are posted by government or non-governmental organization (NGO), commonly receive a wide range of basic preventive and curative care training provided by the government or affiliated NGOs [2]. Informal providers’ practices are usually small and localized, and dependent on the maintenance of good networking and relationships with the communities [2]. Whilst most unqualified allopathic providers and homeopaths provide services through drug shops, CHWs and traditional healers provide door-step services [2]. Because their main responsibility is to deliver health promotion and education, CHWs are not supposed to provide curative care even if they are part of the formal public service delivery [3]. Thus, when they do, their practices become that of informal providers.

Albeit their neglected role, informal providers are vital in bridging the gaps between allopathic and traditional medicine through the services they provide [4]. They constitute a significant component of the health system in most low- and middle-income countries (LMICs), including in Bangladesh [1, 4]. In contrast to the small number of qualified providers, it is estimated that 44% of total health workforce in Bangladesh are traditional healers, 23% are traditional birth attendants (trained and untrained), 8% are village doctors, and 8% are drug sellers [5]. Due to this significant shortage and unequal distribution of qualified providers in the formal health system, people are often left with the option to seek care from informal providers, especially the poor and the disadvantaged [5]. In addition, informal providers regularly serve as the first point of contact for most patients as they share common values and norms, reside close to the community, and thus more accessible and affordable [2, 6, 7]. In rural Bangladesh, around 65% of primary health care is provided by informal allopathic providers [8]. Similarly, the most prevalent form of health care providers in the urban and peri-urban areas appeared to be this type of providers [9].

Clinical practice guideline is a proven tool that can improve the quality of care delivered to the patients [10]. In limited settings like Bangladesh, it offers an aid whereby the health workers adhere to essential practices [11]. In child health, World Health Organization (WHO) established guidelines for preventing and treating diarrhoea and pneumonia among children [12], which have proved to be effective in improving children’s health outcomes [13]. In similar vein, Bangladesh government with the support from WHO created a national guideline for management of hypertension cases, which has been tested and demonstrated to be useful to the primary care physician [14]. Unfortunately, informal providers may not have access to such guideline, leading to incorrect diagnoses and treatment care which may not be appropriate [15].

Appropriate care, defined as “*care that is effective, efficient and in line with ethical principles of fair allocation*” [16] is a concern for health systems across the world [17–21]. Inappropriate care in the form of under-use, misuse and over-use of healthcare services are recognized as a barrier to quality of care that lead to poor health outcomes [22]. In addition, rational use of medicine – *“patients receive medications appropriate to their clinical needs, in doses that meet their own individual requirements, for an adequate period of time, and at the lowest cost to them and their community”* [23], is also a problem in Bangladesh and elsewhere, even in the formal sector [11, 24]. Irrational use of medicine poses serious public health consequences, including treatment failure, increased adverse drug events and accelerating rates of antimicrobial resistance as well as catastrophic health expenditure [25].

Informal providers are significant actors in Bangladesh health system and their role cannot be ignored in the effort to increase effective coverage of health care services, especially in low-income urban settings [9]. It is important to understand their knowledge and practice - how they perceive appropriate care and rational treatment, so they can be more effectively engaged in the delivery of health care services. The current study aims to fill-in this knowledge gap, using qualitative approach undertaken in two peri-urban settings in Bangladesh. However, this is a small-scale study which provides the ground for a further exploration.

## Methods

### Study design

The research employed a qualitative approach to explore the current practice of informal providers and to understand their perception of appropriate care and use of medicine (the two will be referred together as appropriate treatment) for two common childhood diseases in Bangladesh - diarrhea and pneumonia, and a non-communicable disease with increasing prevalence among adults - hypertension. The qualitative study design was chosen to enable profound exploration of informal providers’ perception and barriers to effective care in the light of social, cultural, and physical environment factors surrounding these providers [26].

### Study site and participants

This study was conducted in two peri-urban areas adjacent to the capital city Dhaka, namely Savar and Kamranghirchar. The two areas were selected purposively due to their urbanization characteristics. Savar and Kamrangirchar are currently facing a high industrialization as well as a high influx of rural-urban migration because of expanded employment opportunities. It is assumed that in these settings, informal providers serve as the main providers of healthcare because of the convenience in terms of location and service hours [9].

Although informal providers in Bangladesh comprise of different service providers, a significant number of informal providers in urban and semi-urban areas are unqualified allopathic providers and drug sellers [9]. Therefore, in this study we only included these types of providers, who were also selected purposively. Accordingly, the term informal allopathic providers used in the rest of the paper refers to them.

### Research instruments and data collection

Data was collected using In-depth Interview (IDI). A semi-structured interview guideline was developed by a research team consisting of experienced researchers and graduate research students. The guideline comprised of 34 questions grouped into seven parts to elicit respondents’ perceptions and experiences: socio-demographic characteristics (*n* = 7), health care practices (*n* = 6), perception of appropriate treatment and medicine (*n* = 4), diagnosis process (*n* = 5), clinical guideline (*n* = 4), barriers to deliver appropriate treatment (*n* = 5), and factors to overcome the barriers (*n* = 3). For each question, several probes were created. The perceptions regarding appropriate (rational) use of medicine were explored using questions based on three health conditions with high prevalence in Bangladesh, as explained in the study design section. Before finalizing the interview guideline, a field test was done at a similar peri-urban context. The English version of the IDI guideline was translated into Bangla and back translated into English by the researcher. See the Additional file 1 for the interview guideline. The respondents were approached face-to-face in their workplace and interviewed by two researchers who are the co-authors of this paper. Interviews were audio-recorded with respondents’ permission and each interview lasted for around 30 min. In addition, field notes and reflections were taken during the interviews. In this study, data collection was done until data saturation was reached, when the researcher felt there was no new data obtained from the interviews based on the themes and codes in the interview guideline [27].

### Data analysis

A-priori codes and sub codes were developed before data collection. Transcriptions were prepared from the audio recorded interview immediately following each interview. After data immersion, relevant quotes were subsequently inserted and coded by two researchers in the data display matrix for content analysis - to identify pattern, allow comparison and contrast the findings. Conclusions were drawn guided by the research questions and objectives. Finally, the findings were presented in narrative manner. The current practices of informal allopathic providers in treating diarrhea and pneumonia were compared against WHO guidelines for preventing and treating diarrhea in children [12]. Whereas the treatment practices for hypertension were checked against the National Guidelines of Management of Hypertension in Bangladesh [14].

## Results

A total of 13 IDIs were carried out with the informal allopathic providers, six from Kamranghirchar and seven from Savar. Among the respondents, only one female provider was interviewed. Most providers aged more than 40 years-old with more than 5 years of professional experience. In terms of education, four of them completed tertiary education, seven completed higher secondary (grade twelve) education, and the rest only had secondary education (grade ten). On an average, half of the providers consulted between ten to twelve patients per day, while two of them consulted more than twenty patients daily.

### Current practices

#### Diagnosis practices

Nine out of the thirteen interviewed informal allopathic providers mentioned that knowing the history of the disease and understanding the presenting symptoms were the primary tools for diagnosis. One provider described the process below:

*“As allopathic treatment is based on signs and symptoms, I try to identify the disease mainly based on the symptoms and from my experience. For example, in case of diarrhea, if the stool smells very bad I understand that it is due to food poisoning. Also, I consider patient’s age and the severity of the disease”*. (IDI-1, Section 2.B).

Eight providers also referred to some physical measures such as blood pressure, pulse rate, and body temperature for diagnosis. According to a respondent:

*“I consider few essential things in my diagnosis process and those are- first, the history of the disease and the duration of the disease, blood pressure, body temperature and any other thing that is needed for a particular problem. By this way, I try to understand the severity of the disease. Then I prescribe the necessary drug”.* (IDI-2, Section 2.B).

#### Guidelines followed

Six interviewed providers directly confessed they did not have any guideline from the government or other institution which they followed in their practice. However, they adhered to the written and verbal guidelines and suggestions from pharmaceutical companies’ representatives, even when they did not quite understand the rationale of those suggestions. Meanwhile, four providers were aware of some existing guidelines. Nevertheless, they did not use them, as they felt confident with their ability to diagnose and provide treatment due to their long working experience. Furthermore, four providers mentioned that their ethics were their best guideline, as stated by a new provider who had been in the job for less than 5 years:

*“I do not have any guideline that I can follow. To me, my ethics is my strong guideline in giving medicine and treatment to my patients, as they trust me”.* (IDI-8, Section-2.C).

#### Prescription practice for specific health conditions

In the current study, the providers’ prescription practices were explored for three common health problems in Bangladesh. The practices are described below.

##### Prescription practice for diarrhea

For diarrheal diseases, oral saline or rice-based saline were the commonly prescribed medicine. For childhood diarrhea, the providers considered factors such as the severity, the duration and the volume of diarrhea, as well as the stool’s stench. In addition, they also checked body temperature and observed vomiting propensity. To treat fever, the most commonly given medicine was Paracetamol. Whereas to stop the vomiting, the providers generally prescribed Ondansetron and Domperidon syrup. Eight providers tended to give antibiotics from the first day of treatment. In contrast, only one provider waited for 2–3 days before prescribing antibiotics. Even then, he did it only if there was no demand from the patients to provide antibiotic. Nevertheless, it was also found that two providers never gave antibiotic to children. When their pediatric patients’ condition didn’t improve following symptomatic medication, they would refer them to the hospital. The commonly used antibiotics were Ciprofloxacin, Amoxixilin and Azythromycine. The providers always prescribed the syrup form of these medication for pediatric patients. In addition, all providers also prescribed Zinc syrup for rapid recovery and further prevention of diarrhea. One of the prescription practices by one provider is illustrated below:

*“If it is a normal case, at first I suggest oral saline. But if the patient does not want to take oral saline I give rice saline. Then I give antibiotic either Ciprocin or Quinox syrup (Ciprofloxacin) for seven days, and the dose is one spoon twice a day. Or, I give Napa (Paracetamole) to control the fever. Then, if there is no progress after two days, I give Zox 60 mg (Nitazoxanide). Moreover, sometimes I provide Zinc. However, if the situation does not improve after taking the treatment, I refer (the patients) to hospital”.* (IDI-5, Section 2.B).

##### Prescription practice for pneumonia

In pneumonia cases, it was found that the providers preferred to give antibiotic from the first day of treatment along with medicine to treat fever and cough. The commonly prescribed antibiotics included Cefixime, Cephradine, and Azythromycine. Salbutamol and Prednisolone were prescribed to treat the cough and cold problems. Nebulizer was a common treatment if anyone came with breathing problem, whereas Paracetamol and Domperidon were prescribed to relieve fever and vomiting. If the patient’ condition did not improve after 2–3 days of treatment, they generally referred the patients to the hospital. In addition, three providers mentioned they would increase the medication’ dose. One respondent with over 10 years of working experience stated that:

*“Generally, in this case, I give antibiotic Azythromycine syrup one spoon once a day before meal for seven days. I also give Brodil (Salbutamol) syrup one spoon three times a day for removing cold for five days. Alongside this, I suggest Zinc one spoon three times a day. If this does not work, I suggest going (to) hospital. But if the patient compels me to give more treatment and relies on me, generally I increase the dose of these drugs”.* (IDI-13, Section 2.B).

The interviews as well revealed that two providers preferred injectable antibiotics such as Ceftriaxone. It was also observed that few providers did not want to give any treatment if the patient’s condition was bad. They rather referred the patients to tertiary hospital. Apart from this, one provider would suggest hot drinks and meals, as well as keeping oneself away from dust as an additional care.

##### Prescription practice for hypertension

For hypertension, most patients were asked to continue the medications they had been taking. The most commonly prescribed medicine included Atenolol, Amlodipin, Biosoprolol, Flunarizine, and Flupenthixol. In addition, most providers prescribed other medication such as Diazepam, Bromazepam, and Clonazepam. They decided on the dose and frequency of the medicines’ intake based on patients’ blood pressure. Five providers additionally suggested other preventive and life style modification such as taking less salt, adopting healthy diet and doing more physical activity. One respondent mentioned that:

*“I give medicine based on the degree of severity (of hypertension). If blood pressure is high, I give Amdocal (Amlodipin) and also sleeping pill Tenil or Bopam (Bromazepam). But if it is moderate I suggest Tenorin or Tenoloc (Atenolol). Also, I give some suggestion to follow such as taking less salt or rich food, and to do more physical activity”.* (IDI-5, Section-2. B).

#### Basis for determining medication dose

The prevailing perception among providers regarding dose determination was to consider patients’ age and the anthropometric measure i.e. weight for children. However, only four providers strictly considered age or weight to determine the dose. As a respondent stated:

*“Mainly it depends on both age and weight. But if the age is less than ten years-old, the weight is the main factor to consider”.* (IDI-10, Section 2.A).

Besides this, three providers considered patients’ physical condition, which could be determined by observing the patients. Only one provider with less than 3 years of experience mentioned that disease’s severity along with the duration of the disease was the main factor in determining the dose, as stated:

*“It depends on the age, weight and severity of the disease, but the severity of the disease is the vital factor. For instance, if it is a normal case, in terms of antibiotics, we suggest one spoon. If it is severe we suggest two spoons and if it is really severe we give three spoons, but never more than three spoons for children”.* (IDI-11, Section 2.A).

#### Referral practice

Nine providers mentioned that they would refer patients to specialized doctors or hospital when they could not identify the disease. If the patients did not want to go to the hospital and compelled them to give treatment, which was common, they would provide basic treatment as per their knowledge. Afterwards, if the patient’s condition did not improve or even worsen after taking the treatment for around 5–6 days, they would again refer the patients to the hospital. In case of emergency, all providers would encourage the patients to go to the hospital.

*“Yes, if I cannot manage, I refer to specialist doctor or hospital. When the patient’s situation is severe, I must refer. I even arrange appointments with the doctor that I know over phone sometimes”.* (IDI-6, Section-3).

### Underlying perception and knowledge, and source of information

#### Perception of appropriate treatment

Nine out of thirteen informal allopathic providers reported that correct diagnosis based on history, symptoms and signs as well as appropriate medication were the key elements of appropriate care. Referring to specialist doctors or tertiary hospitals for treatment was another important aspect mentioned, when the disease could not be identified or in case of an emergency. One provider with more than 15 years of experience said that:

*“To know the disease condition correctly by listening to patient’s disease history, making diagnosis and then providing appropriate drugs is the appropriate treatment. If I cannot detect the problem, to refer to the doctor or hospital is also part of my appropriate treatment”.* (IDI-4, Section-2. A).

Five providers also mentioned that identifying the exposure to the disease was the key point in providing the right treatment to patients. Moreover, one provider stated that providing accurate dose of medicine was an important part of treatment to avoid excessive drug prescription. Additionally, few providers mentioned that appropriate treatment could only be ascertained when patients recovered from their illnesses. One respondent with more than 10 years of experience stated that:

*“If patient get well after getting treatment that is appropriate treatment”*.

(IDI-9, Section-2).

#### Knowledge regarding drug (antibiotic) resistance

In the current study, different opinion regarding drug resistance were found among informal allopathic providers. The most common perception was that if a high-power antibiotic was given to a patient initially, a lower-power antibiotic would not work for that patient afterwards. Another prevailing concept was that due to specific immune system’s issue, especially among those who were addicted to recreational drugs or among chain smokers, some drugs would not work or yield good outcome. A respondent with less than 5 years of experience explained that:

*“Due to the specific body’s immune system some drugs do not work, so it is resistance for them. Like, medicine does not bring optimum result for drug addict or chain smoker”.* (IDI-7, Section-2. A).

However, two providers perceived a specific drug to be resistant if after 4–5 days of treatment the condition of the patient didn’t improve. Only one provider considered a sensitivity test to confirm drug resistance.

#### Source of information regarding medication

All interviewed providers in the current study obtained information regarding medication, particularly new drugs, primarily from the pharmaceutical companies’ representatives. These representatives usually provided some written information and sometimes offered seminar or workshop. Some providers established relationship with local registered medical practitioners (Bachelor of Medicine, Bachelor of Surgery/ MBBS doctors) who became their source of information. As mentioned by a respondent with less than 5 years of working experience:

*“I get the information about any new drugs from medical representatives and the leaflets they provide. Moreover, I discuss with one MBBS doctor who comes to my dispensary once or twice a month to pass time with other people”.* (IDI-10, Section 2.C).

Apart from this, the providers also read books and drug guideline. However, they didn’t consider them as the main resource, as most of the materials were only available in English.

### Perceived barriers in appropriate use of treatment

Some common barriers to providing appropriate care and treatment were expressed by all informal allopathic providers below.

#### Lack of training or qualification

During the interview, it was found that all providers received training in the early stage of their career. They mostly received a six-month Local Medical Assistance and Family Planning (LMAF) training. Only one provider obtained a Diploma in Medical Faculty (DMF), a four-year course which includes an internship. These courses offer a basic training on health care and medicine. The providers considered the training as important because it gave them the only valid way to enter their profession. Furthermore, the training helped to raise their confidence in treating patients and facilitated networking with other participants. However, the providers expressed that the training duration was too short to enable them to obtain the necessary knowledge or to qualify them for more specific treatments. In addition, they complained that there were several institutions offering LMAF training which did not maintain their training quality. A provider acknowledged that:

*“I am lacking the training that will give me the skill to treat patient appropriately. I also cannot give treatment for many conditions such as for pregnant women, as I did not have the specific training for this. But besides that, my degree also does not permit (to treat pregnant women)”.* (IDI-3, Section 3.A).

The insufficient training that providers received has influenced negatively on their knowledge. Some providers confessed that sometimes they could not understand the pathological (diagnostic test) report and the prescription from MBBS doctors. They did not have any other way to understand the prescription without asking the medical representatives, and they could do it only if they have good relationship with them.

#### Lack of diagnostic tools and medicine

To provide appropriate care, the providers recognized the importance of diagnostic tools that can help them to make proper diagnosis. Nevertheless, most of them did not have the access to the required diagnostics. A provider with more than 15 years of experience pleaded:

*“We need modern diagnostic equipment so we can make proper diagnosis”.* (IDI-5, Section 3.A).

In addition, sometimes they also did not have the right medicine because they did not have the permit to store and sell certain drugs. A respondent said that:

*“Sometimes I cannot give the drugs according to the prescriptions as I cannot (not allowed to) keep certain drugs”.* (IDI-8, Section-3. A).

#### No systematic and efficient regulation system

In Bangladesh, there is no established regulation system to monitor informal providers’ practices, let alone their quality of service. For most providers, no one ever checked their qualification and their license. Only two providers mentioned they faced a mobile court once in the course of their profession, which was more than 10 years ago. One respondent mentioned:

*“I never see any kind of monitoring body to supervise our work or check whether our license is valid or not during the time that I have worked”.* (IDI-9, Section-3. A).

However, another provider argued that penalizing them for running their practice was not an effective way to address the issue of appropriate treatment, as agreed by another provider:

*“Indeed, there is no gain to take any kind of hard step against informal providers. Rather, giving motivation is important to increase our capability for providing proper treatment”.* (IDI-9, Section-3. A).

#### Patient pressure to provide specific medicine

Eight providers stated that it was common for the patients to demand some medicine according to their choice. Since going to a specialist doctor would require a lot of money for the diagnostic tests and consultation fees, many patients urged the providers to treat them instead. In addition, the patients usually wanted to get well quickly, thus they requested for antibiotics from the beginning, and sometimes even for a higher dose. A provider with less than 15 years of experience explained that:

*“Few patients asked for antibiotics as they believe that by taking antibiotic the recovery will be quick; they sometimes even mentioned the name of a specific antibiotic. In such cases, it is tough for us to resist patient’s request, although we know this might not be good for their health”.* (IDI-13, Section 2.C).

Because of their business orientation, the providers did not want to lose any patient. Therefore, two of them usually complied with patient’s request. Nonetheless, two providers mentioned they also tried to convince patients to trust them with the diagnosis and treatment. Fortunately, one provider mentioned that patients’ tendency to give them pressure was decreasing day by day.

### Suggested ways to overcome existing barriers

#### Enforcing regulation and monitoring

Five providers believed in the importance of regulation enforcement to make them more accountable. They suggested to establish a strong regulation system with regular monitoring activities, so that uncertified and unlicensed providers could be disciplined. They also argued that similar regulation and monitoring should be applied to pharmaceutical companies as well. They indicated that many pharmaceutical companies operate outside of the regulation system. A provider with between 15 to 30 years of experience explained that:

*“It is really tough to overcome many existing problems. There are many pharmaceutical companies which are established and operate without maintaining the rules and regulation”.* (IDI-1, Section 3.B).

#### Comprehensive training and refresher training

The providers recommended that area-based training by a skilled health professional known to the providers, preferably an MBBS doctor, could be a way to improve their knowledge and skill. This area-based training was suggested because it would be more acceptable to the providers, especially in terms of accessibility. The same respondent who has between fifteen to 30 years of experience suggested:

*“It would be better to arrange area-based training by skillful persons like MBBS doctors”.* (IDI-1, Section 3.B).

The providers also urged LMAF program to update training curriculum and to increase training duration. Two providers mentioned that many patients visited them for pregnancy-related care. However, they could not offer the service because of their lack of knowledge. Therefore, they requested for a special training that can help them to meet these demands in the future.

#### Recognition by the health system and the authority

A common expectation expressed by the providers was a proper recognition and acknowledgement of their important role in the health system. They realized that they serve a large proportion of the population without imposing expensive user charges. The current situation which did not recognize them as an integral part of the system made them feel unsettled and unmotivated to improve their capacity and practice. A respondent pleaded:

*“Do not exclude us because we are village doctor. Rather consider us as important helpers who are trying to give the right treatment to patients. Please create some good policy for us where we can get the recognition”.* (IDI- 2, Section 3.B).

They proposed that establishing an effective informal allopathic providers association in collaboration with government body could be a good approach to ensure their accountability. As voiced by one provider with less than 5 years of experience:

*“Establish effective informal providers association in collaboration with government body, and include all informal providers for ensuring accountability”.* (IDI-10, Section-3. B).

#### Making the literatures and guidelines available in Bangla

The providers revealed that language was a barrier to access the information and other written materials provided by pharmaceutical companies. They suggested the materials to be offered in Bangla, so they can understand properly how the medicine works, the appropriate use and dose, as well as the side effects. They also recommended that the material can be designed in a way that is easier to understand, so the providers would not hesitate to refer to them when necessary. One respondent argued that:

*“It would be more effective for us if we get all the clinical guideline in Bangla (language) and in an easier form so that we can consider these materials as a useful resource in our profession”.* (IDI-2, Section- 3.B).

#### Encouraging informal providers to consider patient safety

The informal allopathic providers mentioned that maintaining the ethical aspect during treatment was important for appropriate care and treatment. Due to their business orientation, sometimes the providers prescribed medicine that was not needed and even harmful to the patient. In their opinion, this tendency could only be avoided if ones followed ethical principles. One provider with less than 15 years’ experience mentioned that:

*“...However most important factor is our own ethical concern to stop wrong treatment and misuse of medicine”.* (IDI-13, Section 2.C).

#### Educating patient

Two providers highlighted the vital role of patients to choose which provider to go to for their treatment. They considered it to be beneficial for the patients to understand the performance of different providers, so they can trust the treatment of the provider of their choice. For the providers themselves, being identified as a good provider would give them the encouragement to treat their patients more effectively and efficiently. Another respondent with less than 15 years’ experience mentioned that:

*“I think it is equally important to educate patient on the appropriate treatment and inform them regarding the side effect of antibiotic if it is not necessary, as many patients insist to receive antibiotic from the first day, even if it is not required.”* (IDI-3, Section- 3.B).

## Discussion

The current study explored the perception of informal allopathic providers in two peri-urban areas in Bangladesh on appropriate care and use of medicine along with the existing barriers to improve their practices and pathways to overcome these barriers. This study found that their perception varied, with some providers already acquired good knowledge. However, many of them still have erratic ideas about appropriate treatment. This variation might be due to their different backgrounds, work experience, as well as the knowledge and insights they gathered from their work. Previous studies comparing informal allopathic providers with formal health providers indeed demonstrated that informal providers’ knowledge and practices are more varied, with their lack of proper training cited as an important underlying factor [8, 15, 28].

### Current practices and their implication

According to WHO guideline for preventing and treating diarrhea in children, diarrhea treatment includes advising and teaching mothers or care-givers to increase the amount of fluids intake and to continue feeding until the condition stops [12]. However, this simple, easy and appropriate practice was not mentioned by any of the respondents. In addition, as per WHO guidelines antibiotic should be prescribed only in the case of bloody diarrhea, with Ciprofloxacin as the recommended choice of drug [12]. Nonetheless, in practice other third-generation antibiotics, such as Cefexime and Azythromycine were prescribed by the majority of providers. Based on the guideline, the provision of vitamin A for children from 6 months to 5 years old, and the administration of oral saline solution (ORS) with Zinc supplement are recommended [12]. Nevertheless, no provider interviewed provided vitamin A, although ORS and Zinc supplement administration were common.

Based on the same WHO guideline, for children who are suffering from mild pneumonia, Amoxicillin is the recommended choice of drug to be given for 5 days [12]. For severe cases, parenteral Ampicillin or Penicillin (if Ampicillin is not available) together with Gentamicin for 10 days is the recommended treatment [12]. For children more than 1 year old, Cotrimoxazole can be used only when clinically indicated [12]. In this study, it was revealed that no provider provided Ampicillin to treat pneumonia in children. Instead, the common practice was to treat using third-generation antibiotics such as Cefexime and Azythromycine syrup and antibiotic injection administration such as Ceftriaxone.

The over-use and misuse of antibiotics in the current study for both diarrhea and pneumonia is concerning, as the WHO guideline clearly outlines that antibiotics are to be used only for severe cases [12]. In addition to treatment failure, the rapid development of drug resistance and poor health outcomes, this practice can cause a dire consequence of calamitous additional health care costs [25]. The providers might decide to give antibiotics due to their lack of knowledge. As reflected in this study, the providers were not well-aware of the side effects of antibiotics; they only had limited knowledge regarding drug resistance. Another issue with antibiotics provision is associated with the providers’ business orientation. Because informal allopathic providers do not usually charge or charge a little for consultation, their livelihood depend largely on the drug sale. The provision of antibiotics, including the third-generation antibiotics, might be due to their attempt to satisfy patients’ demand or to provide medicine that can cure the illnesses quickly, in fear of losing the patients otherwise [29]. Indeed, the interviews in this study revealed that providers sometimes received pressure from their patients to give antibiotics. Similar finding was demonstrated by a previous study from Bangladesh, that the rate of inappropriate use of medicine including antibiotic was higher among informal allopathic providers [2]. The prescription of multi-drug, unnecessary expensive drugs and injections were common especially among drug sellers [8]. Additionally, unqualified allopathic providers are known to be lacking the knowledge on the dose and side effects of the medicines they prescribe [30]. Nonetheless, the providers in this study seemed to have good knowledge on how to determine the dose by considering important factors such as age, weight, and the severity of disease.

With regards to hypertension, a previous study reported that around 13.5% of adults in Bangladesh suffered from hypertension [31], with around 41% being diagnosed and treated by informal allopathic providers, leading to a higher rate of non-adherence to medication [32]. According to the “National Guidelines of Management of Hypertension in Bangladesh”, hypertension diagnosis is based on blood pressure measurements on several occasions and the treatment is based on three principles: medical history, physical examination and laboratory investigation (urine analysis, blood glucose testing and a 2-led ECG report) [14]. There are several types of hypertension including accelerated, secondary, pregnancy and childhood hypertensions which may require a range of treatment from non-pharmacological therapy only to a specialist’ care [14]. In this study, the providers provided not only anti-hypertensive drugs but also sleeping pills. Without knowing the type of hypertension and understanding the potential adverse effects of the medication, these practices can be harmful. Nonetheless, it was encouraging that most providers considered other preventive and life style-based treatment for the patients.

The failure of informal allopathic providers to provide appropriate treatment can lead to a haphazard referral and delay in health care seeking [33]. Most providers in the current study would refer the patients when their conditions were severe or when they could not identify the problem. However, one finding to be highlighted is the fact that the patients often gave pressure to the providers to treat them, even when they have been advised to seek care at a tertiary hospital or a specialist doctor. This happened mainly due to financial issues, as seeking care in the hospital or with a specialist doctor can entail expensive spending for diagnostic tests and consultation fees. Previous study has pointed out the preference of poor populations to seek care in informal providers, amongst others because of affordability issue [34]. However, the current study found that providers also failed to give appropriate treatment because they did not have the right diagnostic tool or medicine.

### Perceived barriers and suggested ways forward

Some studies have suggested that regulatory systems have been ineffective in Bangladesh to monitor the sales and practice of informal allopathic providers especially in the rural areas [35]. Although they receive a high demand for their service, they have very little contact with the government to get the support they need or to be accountable to [36]. In Bangladesh context where a large proportion of the population rely on informal allopathic providers, it will be more realistic to acknowledge their role and to integrate them into the system. In this manner, the authority can engage them more effectively in the activities to achieve public health goals by building on their motivation and ethics. Considering the slow production and recruitment process of the formal health workforce in Bangladesh [37], engaging informal providers might be helpful in achieving the government’s goal of universal health coverage (UHC) by 2030. To integrate them into the health system, however, would need to consider the opinions of different stakeholders, including the authority, other qualified allopathic providers, health officials and potential clients. Nonetheless, similar suggestion was also made by a study from Nigeria, which investigated the self-described roles and motivations of informal providers in rural part of the country [38]. Indeed, the providers interviewed in this study saw themselves as part of the health sector, therefore punishing them for their practice was not considered a wise approach. Several studies in the past have assessed the effectiveness of engaging informal allopathic providers, either paid or unpaid, to increase access and coverage of basic health services, especially for tuberculosis and childhood fever [39]. These studies suggested that their engagement have resulted in improved diagnosis, treatments, and referrals for those diseases [39].

A few studies also highlighted that effective training can play a significant role to improve informal providers’ capacity in providing primary care and proper treatment [40]. In the current study, all providers acknowledged the importance of adequate training. They expressed the need for refresher or additional training. A previous study also recommended that informal providers should receive a basic pharmaceutical training [30]. These can be organized through informal allopathic providers association and arranged locally based on their service area, as the providers themselves suggested. It has been previously proved that providing intensive training, supplying providers with clinical management guideline and accreditation of provider’s outlets significantly improved informal provider’s practices [4]. In Vietnam, face-to-face training coupled with regulatory enforcement to decrease the sale of antibiotics without prescription was also proved to be effective [4]. This type of training, along with provision of clinical guideline in Bangla language might be effective in Bangladeshi setting. A training around some specific guidelines that informal allopathic providers are required to follow can be one of the approaches. Guideline can serve as a simple tool to instruct providers to perform certain procedures or practices [11, 41]. This can improve providers’ confidence in clinical decision-making as well as their practical skills and attitude to care [41]. The need for guideline communicated by some providers in this study might be the expression of their lack of confidence due to their limited knowledge and skills, and their wish to overcome it. A lack of basic knowledge on how to diagnose and manage common diseases is undeniably associated with suboptimal, ineffective and dangerous health care practices [41].

It is also increasingly recognized, however, that providing case management guidelines and training alone are not sufficient to sustain good performance of health workers [42]. It is suggested that combining training with other mechanisms, such as supervision or group problem solving, or implementing certain multifaceted strategies i.e. the combination of training, supervision, printed materials for health workers and patients, providing equipment and supplies including diagnostics, accreditation, and establishment a referral network [43]. There is a potential to involve government in this effort, as well as to engage medical professional associations, pharmaceutical companies and NGOs to supervise and monitor their practice in a supportive manner. All of these efforts require substantial commitment and investment by the authority. Nonetheless, if informal allopathic providers become the integral part of the health system, the use of guideline and other patient safety measures can be established, enforced and monitored more effectively.

Finally, engaging and educating patients is equally critical. As discovered in this study, the providers indicated the crucial role of the patients in identifying a good provider to seek care from. This in turn can be utilized as an incentive for informal providers to improve their practice in order to attract the clients. Different ways of effective patient education in the past were undertaken by taking into consideration the primary language spoken by patients, their culture, gender, ethnicity, education background, general literacy and health literacy, as well as the available resources in the community. Furthermore, having a provider that patients can trust will facilitate their acceptance of provider’ therapeutic recommendations, adherence and satisfaction with the recommendations, leading to improvement in health outcomes [44]. Unfortunately, there has not been much work done to engage and educate patients in LMICs with regards to primary health care service, and this can be an area for future research.

The results of the current study may not represent all informal allopathic providers throughout Bangladesh, as the study comes from limited study areas and respondents. This study serves as an exploratory study, and a future mixed-method study with bigger and more diverse sample will provide a better picture of their scope and practices in Bangladesh. Because of time and financial constraints, only qualitative approach was conducted in this study. Additionally, the opinions of other relevant stakeholders would enrich the findings. Finally, as self-reported information was collected from the participants, there might be a chance of recall and information bias; the respondents might be led to give a favorable or correct answer.

## Conclusions

Unqualified allopathic providers lacked the knowledge and skills for delivering appropriate treatment and care. Their services did not always confirm to best practices, and several barriers existed that prevent them from delivering appropriate treatment. An important way to tackle this would be to acknowledge their role in the health system and then legitimately offer different intervention to address the factors that affect their practices. Being the providers for a substantial proportion of the population, it is crucial that policy makers become cognizant of the fact and take measures to remedy the issues around informal providers’ practices. This is even more urgent if government’s goal to reach UHC by 2030 is to be achieved. As the sample size of the current study is small, the results can be considered as preliminary findings that can guide future larger studies. The opinions of other stakeholders are essential to create sound and feasible policy recommendations.

## Additional file

Additional file 1: Participant informed consent form and in-depth interview guideline. (DOCX 55 kb)

## Data Availability

The dataset generated and analyzed during the current study is stored in the James P. Grant School of Public Health’ data repository and is available on a reasonable request to James P. Grant School of Public Health’ authority (Director of Research, Dr. Malabika Sarker, at malabika@bracu.ac.bd).

## Abbreviations

CHW: Community Health Workers
DMF: Diploma in Medical Faculty
IDI: In-Depth Interview
JPGSPH: James P Grant School of Public Health
LMAF: Local Medical Assistance and Family Planning
LMIC: Low and Middle-Income Country
MBBS: Bachelor of Medicine, Bachelor of Surgery
NGO: Non-Governmental Organization
UHC: Universal Health Coverage
WHO: World Health Organization
