# Supplementary material for: Perceptions of appropriate treatment among the informal allopathic providers: insights from a qualitative study in two peri-urban areas in Bangladesh
Source: BMC Health Serv Res. 2019 Jun 26;19:424. doi: 10.1186/s12913-019-4254-3 (PMC6595608; doi:10.1186/s12913-019-4254-3)
Supplement: Supplementary file 1 — Participant informed consent form and in-depth interview guideline. (DOCX 55 kb) [file 12913_2019_4254_MOESM1_ESM.docx]

participant INFORMED CONSENT FORM

in-depth Interview

**G**eneral objective: To study whether the informal providers in Bangladesh can reduce harmful practices through rational treatment and use of medicine.

**G**reetings, I am Monaemul Islam Sizear and this is *(If there are two interviewers)*. I am/we are working in an academic research project under James P. Grant School of Public Health, BRAC University.

We are conducting a study on the informal providers (drug sellers, village doctors, homeopathic doctors, folk healers etc.) in Bangladesh. The general objective of this study is understanding whether they can reduce harmful practices through rational treatment and the use of medicine. In this study, we also wish to explore the disease and patient profile, harmful practices according to their knowledge; how they receive information on medicines (side-effects, contraindications or dangers of inappropriate use), from whom and to what extent; their diagnosis, prescription and treatment process; the business model and money making process and their motivation for being in the business; and perception regarding ‘rational treatment of diseases’ and ‘rational use of medicines’ and to identify their perceived barriers in implementing rational treatment as well as how they can overcome it.

We invite you to participate in this study. You will have to answer some questions in the subject explained above. The interview will not take more than 40-45 minutes. Your responses will be kept confidential and will not be used for any other purpose other than this study. You can ask for any clarification of any question and can withdraw from the study anytime you want.

Do you have any further inquiry about this study? Yes No

(*If “Yes” please answer to any other inquiry of the parent/ guardian)*

Do you agree to participate in this study? Yes No

**In-depth Interview Guideline**

**Objective:**

To explore the perception of informal health care providers (mainly allopathic practitioner) regarding ‘appropriate treatment of diseases’ and ‘rational use of medicines’ and to identify their perceived barriers in implementing rational treatment as well as how they can overcome it.

| **Interview details** | |
| --- | --- |
| **Date:** |  |
| **Code (ID):** |  |
| **Interview Start Time:** |  |
| **Interview Finish Time:** |  |
| **Interviewer:** |  |
| **Note-taker/Tape Recorder:** |  |
| **Name of Village:** |  |
| **Name of Ward:** |  |
| **Name of Thana (Sub-district):** |  |
| **Name of Zila (District):** |  |

**Instructions for interviewers:**

- Check recorder before starting interview.
- General question earlier and specific question later
- Non-threatening question first.
- Structured sensitive question later.
- Don’t ask one more question at a time.
- Don’t ask any leading question.
- Don’t use any jargon or medical terminology.

| **Participant Details** | |
| --- | --- |
| **Name:** | |
| **Age:** | **Gender:** Male Female |
| **Marital Status:** Married Unmarried Divorced Widowed | |
| **What is the highest level of formal education that you have completed?**  No Education Primary (5^th^ grade) Secondary (10^th^ grade) SSC  HSC Undergraduate Post-graduate Madrasa Others | |
| **What is Your Religion?**  Islam Hinduism Christian Buddhism Others | |
| **What is Your Ethnicity?** Bengali Other | |

| **1. General Background** | | |
| --- | --- | --- |
| **Question** | | **Probe** |
|  | Tell me something about your profession? | - How long have you been in this profession?  - Where do you practice? |
|  | Why did you become a healer/ doctor? | - What motivate you to be a health professional?  - Do you have any family member who also work as a health professional? |
|  | What type of treatment do you provide to people and what are they? | - Allopathic/Herbal medicine?  - Can you tell us the name of diseases that you treat?  - Diarrhoea  - Pneumonia  - Hypertension |
|  | Number of patient visited you every day on average? | - For which disease do patients usually come?  - What do you think, why people come to you?  - Why do they trust you? |

| **2. Perception towards appropriate use of medicine and treatment** | | |
| --- | --- | --- |
| **Question** | | **Probe** |
|  | How do you perceive the idea of “appropriate treatment” (good, ideal, appropriate treatment)? | - Can you explain about ideal/good treatment as you see it?  - Can you tell me the importance and benefits of appropriate/good treatment?  - What are the characteristics (dose, duration of treatment, medical history) of good treatment? |
|  | What is your diagnosis process?  **Scenario 1:** A female two years-old child had 4-5 bouts of watery diarrhea since morning. She has slight fever and vomited once. She is refusing usual food. | - What are the specific things that you do to find out about problem?  - How do you reach a diagnosis?  - What treatment would you advise or prescribe? |
|  | **Scenario 2:** A male child aged 3.5 years-old had cough and fever for past two days. Now he is running high fever with rapid breathing. He seems to be lethargic and have stopped taking any food. | - How you will reach a diagnosis?  - What treatment you would advise or prescribe? |
|  | Are you following any guideline to prescribe/give medicine? | - What are your source of information about medicine?  - Do you have any kind of standard of treatment in case of diarrhoea, pneumonia and hypertension?  - Do you have any booklet that you follow?  - Do you have you got any written materials from drug company/pharmacy?  - Is there any pressure from patient to give particular medicine as their demand? |

| **3. Barriers in Implementing appropriate Use of Medicine & Treatment & Overcoming Pathways** | | |
| --- | --- | --- |
| **Questions** | | **Probe** |
|  | Do you face any kind of problem in your service to give appropriate treatment and medicine? | - Can you think of some barriers (e.g. personal, knowledge gap, medical equipment’s, human resource, socio-economic, political)?  - Do you have sufficient equipment to give proper treatment as you need?  - How do/did you overcome these/those barriers? |
|  | Have you ever received any health-related basic training or medical training? | - From where did you receive your training?  - Duration of training?  - How many training have you taken?  - How effective a training can be to give better treatment? |
|  | Do you refer to any other health professional if you cannot detect the problem of patient | - Do not you think it is better to refer to others if you cannot understand properly the problem?  - If a patient did not get well after taking long time treatment from you, do you suggest any other places?  - To whom usually do you refer? |
|  | Has anyone supervised/regulated your service in any time? | - Has any government or non-government body visited to see your work?  - Have you ever received any kind of assistance from government and NGOs that could be helpful for your job?  - To whom are you bound to explain about your given treatment? |
|  | How do/did you overcome these/those barriers | - What is your suggestion to solve these problems as you mentioned?  - Can you tell me the best way to solve the problem? |
|  | Do you feel there is something important we should have asked that we haven’t? | - Would you want to add anything else?  - Can you give me your comments about the matter I asked you in my questions? |

***[Thank you very much for your time]***
